# Pregnant adolescents’ dynamic engagement with participatory women’s groups for maternal and newborn health in rural India: a qualitative study

**DOI:** 10.1136/bmjph-2024-001309

**Published:** 2025-08-14

**Authors:** Farnaz Sabet, Smita Dattatraya Todkar, Nirmala Nair, Susan M Sawyer, George C Patton, Suchitra Rath, Audrey Prost

**Affiliations:** 1 Royal Childrens Hospital, Centre for Adolescent Health, Parkville, Victoria, Australia; 2 Population Health Theme, Murdoch Children’s Research Institute, Parkville, Victoria, Australia; 3 Paediatrics, The University of Melbourne Melbourne Medical School, Melbourne, Victoria, Australia; 4 EKJUT, Ranchi, Jharkhand, India; 5 Child Development Group, Sangath, Porvorim, Haryana and Goa, India; 6 Centre for Doctoral Studies, Manipal Academy of Higher Education, Manipal, KA, India; 7 University College London Institute for Global Health, London, England, UK

**Keywords:** Community Health, Public Health, Sociodemographic Factors, Humans

## Abstract

**Introduction:**

Around 21 million girls globally become pregnant each year, many in the context of early marriage, yet we know very little about supporting them during the perinatal period. This study explores how pregnant adolescents engage with women’s groups practising participatory learning and action (PLA) to improve maternal and newborn health in rural eastern India.

**Methods:**

The study was carried out with Ekjut, a non-governmental organisation responsible for training government-incentivised volunteers who facilitate groups in several Indian states. A team of three qualitative researchers carried out 29 semistructured interviews, nine focus group discussions and five video-recorded observations of groups in 12 villages of Jharkhand, eastern India, from December 2018 to December 2019. We interviewed girls and women with different levels of engagement with PLA groups, as well as health workers and analysed data using thematic analysis.

**Results:**

Pregnant adolescents wished to attend PLA groups and learn from them but faced many barriers to doing so. These included restrictive marital norms that were stronger with younger age and enforced by pervasive gossip. Marital family support and access to a skilled government health worker, however, could moderate these restrictive norms to enhance engagement with PLA groups. Pregnant adolescents expressed wanting elders present in PLA groups and valued marital family support to help them navigate the challenges of pregnancy and motherhood. Pregnant girls from more restrictive families were particularly vulnerable and socially isolated, requiring more intense engagement from health workers to connect them to PLA groups and health services.

**Conclusion:**

Research and programmes to support pregnant girls would benefit from acknowledging the restrictions of early marriage and pregnancy while also engaging constructively with these restrictions. This might involve recognising the potential of PLA groups to foster adolescents’ developmental capacities but also constructively engaging with marital families and providing broader developmental support.

**WHAT IS ALREADY KNOWN ON THIS TOPIC**

- Around 13 million adolescent girls give birth annually in low- and middle-income countries, yet evidence to support their maternal healthcare needs is missing. Most perinatal health interventions do not consider adolescent-specific needs, nor do they assess how these interventions work for them.

**WHAT THIS STUDY ADDS**

- This is one of a very limited number of studies to explore pregnant adolescents’ engagement with a perinatal health intervention from their own perspective. It is also the only study that explores how pregnant adolescents engage with an intervention included in WHO’s recommendations for addressing neonatal mortality in low-resource settings with high mortality: women’s groups practising participatory learning and action to improve maternal and newborn health.

**HOW THIS STUDY MIGHT AFFECT RESEARCH, PRACTICE AND POLICY**

- The study offers broad insights for supportive programming for pregnant adolescents in the context of early marriage. We found that considering pregnant adolescents’ developmental needs in addition to their perinatal needs could help boost their engagement with PLA groups. Girls wanted supportive elders and family guidance and wished to develop their capacities, particularly through learning and peer friendships. We suggest that these adolescent-sensitive developmental considerations should inform interventions that include pregnant adolescents.

## Introduction

 An estimated 13 million girls give birth annually, with almost one million of them in India.^1^ Pregnant adolescents, defined as those aged 10–19 years, face multiple risks and disadvantages.^2^ These include higher maternal mortality compared with women aged 20–29 years old,^3^ as well as higher rates of neonatal mortality, preterm delivery and low birth weight.^4^ Developmentally, pregnant adolescents have specific physiological,^5^ psychological and social needs.^6^ Many also face power dynamics and restrictive social norms^7^ that result in barriers to care and reproductive decision-making, particularly in the context of early marriage.^7 8^

We know little about supporting pregnant adolescents in low- and middle-income countries. A recent systematic review found a striking lack of research on interventions to improve outcomes among pregnant adolescents and their newborn infants in these settings.^2^ This lack of adolescent-specific research reflects an assumption that pregnant adolescents have similar needs to pregnant adults and that community-based interventions offered to all pregnant women in a community will also reach and support pregnant adolescents. Moreover, given adolescent engagement with healthcare is generally poor,^9^ it is important to assess if perinatal healthcare interventions in areas of high adolescent fertility rates also adequately engage adolescents.

One intervention that includes pregnant adolescents and is rolled out in low-resource settings with a high burden of neonatal mortality is participatory learning and action (PLA) with women’s groups. PLA with groups is a community mobilisation intervention where participants come together in monthly meetings with a trained facilitator. Meetings follow four phases and allow groups to: identify and prioritise common perinatal health problems in their community; collectively discuss strategies of how to respond; put the strategies into action and evaluate their effects.^10^ Groups typically engage the wider community in understanding and supporting their strategies through two large community meetings.^11^ A meta-analysis of seven trials of the intervention in Bangladesh, India, Malawi and Nepal found a 20% reduction in neonatal mortality.^12^ Two efficacy trials from the Indian state of Jharkhand found even larger (30–32%) reductions in neonatal mortality.^13 14^ Subsequently, India’s National Health Mission issued a recommendation advising that PLA with women’s groups to improve maternal and newborn health be scaled up across 10 states with high rates of neonatal mortality, such as Madhya Pradesh and Bihar.^15^ Many of these states also have high rates of adolescent pregnancy within the context of early marriage.^16^ Our study aimed to explore the extent to which pregnant adolescents were able to engage with PLA groups that were scaled up through the National Health Mission in the state of Jharkhand, using qualitative methods.

## Methods

### Setting

Jharkhand is a largely rural state in eastern India. Around a quarter of the state’s population belongs to indigenous communities (Scheduled Tribes (STs)).^17^ Although early marriage, under 18 years for women and 21 years for men, is prohibited in India as a whole, ^18 19^ the prevalence of early marriage in girls was 31.5% in Jharkhand in the 2021 National Family Health Survey.^16^ Despite improvements in the last two decades, Jharkhand’s maternal mortality ratio also remains high, at 56 per 100 000 livebirths,^20^ as does the neonatal mortality rate, at 32 per 1000 livebirths.^21^ Rural areas of Jharkhand have one of the highest adolescent fertility rates in India, at 73 per 1000^16^ (compared with 43 per 1000 nationally) with most adolescent pregnancies occurring within the context of early marriage.

This study was developed with Ekjut, a non-governmental organisation with a 20-year history of supporting participatory women’s groups.^13 22^ From 2017 to 2019, in collaboration with the National Health Mission, Jharkhand, and University College London, Ekjut conducted a non-randomised controlled evaluation of PLA with women’s groups scaled up by government-incentivised frontline workers known as Accredited Social Health Activists (ASHAs or *Sahiya* in Hindi).^23^ We used the systems and relationships in place from this evaluation to conduct our study.

### Study design and sampling

To explore pregnant adolescents’ engagement with PLA groups, senior researchers from Ekjut identified stakeholders whose perspectives would be informative. We wished to explore the perspectives of adolescents who had different levels of exposure to PLA groups: those who had attended groups, those who lived in areas with PLA groups but had never attended and those who lived in areas where PLA groups had not yet begun. We also wished to tease out the unique perspective of being a pregnant adolescent and differentiate this from the experience of simply being an adolescent or having a first pregnancy. We therefore also interviewed unmarried, non-pregnant girls who attended PLA as well as primigravida adults. Additionally, Ekjut’s experience suggested that family members’ influence on decision making for their daughters-in-law’s pregnancies was significant and that their views on PLA groups were important to capture. Interviewing family members as well as healthcare providers across primary, secondary and tertiary care in Jharkhand was also strongly encouraged by the local ethics committee, who advised that this perspective was critical to inform any resulting change in programming of PLA groups to better support pregnant girls. Although PLA with groups is a community intervention, interviewing health providers at secondary and tertiary levels, including Adolescent Sexual Reproductive Health workers, would give insights into the engagement of more vulnerable cases, such as those who had suffered a recent perinatal loss or experienced severe family violence which our distress protocol excluded.

The authors (FS, SDT, SR) piloted interview guides in two different villages and iteratively adapted the topic guides and methodology, adding for example questions on mental health, family violence and alcohol use (see online supplemental file 1 for interview guides). Patients and the public were not involved in the design, conduct or reporting of the study, but we plan to involve them in the dissemination of findings.

The authors (FS, SDT, SR) recruited adolescent girls and their families through ASHAs. ASHAs normally follow-up all pregnant women and adolescents who present for antenatal care in their catchment area (usually a village). Ekjut knew many ASHAs as they were responsible for training them to run PLA groups. We visited 12 villages and, together with ASHAs, identified all adolescents (aged 19 years and under) who were pregnant or already mothers, using the ASHAs’ antenatal care lists. Drawing on the ASHAs’ knowledge of their communities, we excluded very vulnerable girls (see online supplemental file 1) and purposively sampled participants to capture different ages, levels of schooling, varying exposure to PLA groups and caste or tribe backgrounds (Scheduled Tribe (ST), Scheduled Caste (SC) and Other Backward Class (OBC)). Depending on the numbers of pregnant and mothering adolescents in a village and their availability, interviewers in consultation with ASHAs determined whether that village was more suited for conducting focus group discussions (FGDs) or interviews or both. FS and SDT recruited health workers by directly approaching them in hospitals and clinics across different levels of health provision in Jharkhand from the village to the district level. We adopted a snowball sampling method: those we interviewed suggested others to interview.

### Data collection

All who were approached consented to participate. In total, we conducted 29 individual interviews and nine FGDs with 104 unique participants, of which 26 were pregnant adolescents or adolescent mothers. We also videorecorded five PLA group meetings with approximately 175 participants. The interviews included 12 pregnant adolescents, an additional three adult primigravida women, three nulliparous adolescents and one husband of a pregnant adolescent (as no other family members were present to form an FGD). Three FGDs were arranged for family members living with pregnant adolescents interviewed, another three FGDs were arranged for pregnant or mothering adolescents, two for nulliparous adolescents and one FGD for ASHAs. Finally, our study included 27 health workers (10 interviews and 17 in the ASHA FGD). We encountered no distress in interviews or FGDs. Table 1 describes participant characteristics in more detail.

**Table 1:** Participant characteristics

	Semi-structured interviews	Focus group discussions (FGD)
**Pregnant adolescent attending PLA** **and resident family**	**5**	**3 FGDs of family members (11 participants)**
	Girl A : adolescent OBC primigravida	Family of A: father-in-law, mother-in-law, brother-in-law
	Girl B : adolescent ST primigravida	Family B: husband
	Girl C : adolescent ST mother (death of child as a neonate)	No family available
	Girl D : adolescent OBC mother	Family D: sister-in-law of mother-in-law and her two daughters
	Girl E : adolescent ST primigravida	Family E: father-in law, mother-in-law, sister-in-law, younger brother-in-law
**Pregnant adolescents or adolescent mothers (n=26)**	**12**	**3 FGDs of adolescent girls** ^*^ **(33 participants)**
Age (years)		
15	2	0
16–17	4	2
18–19	6	12
Social Class		
ST	6	9
SC	3	3
OBC	3	2
Schooling attained		
<9 th class	3	6
9–10th class	5	2
11–12th class	3	0
College	1	0
Not collected	0	6
Marital status		
Married	12	13
Number of pregnancies		
1	9	9
2	3	5
Neonatal loss		
0	11	13
1	1	1
Number of PLA sessions attended		
0	7 (4 in areas without PLA)	4
1–3	3	7
4–5	2	2
≥6	0	1
**Nulliparous adolescent girls**	**3**	**2 FGDs of nulliparous adolescent girls (39 participants)**
Age (years)		
11–14	1	7
15–17	2	20
18–19		12
Social class		
ST	1	11
SC		16
OBC	2	6
Not given		6
Schooling attained		
≤6 th Class		3
7–9th Class	1	14
10–12th Class	1	13
College	1	6
Not collected		3
Attendance at PLA		
Yes	3	26
No	0	13
**Adult primigravidae**	**3**	
Age (years)		
Range	21–24	
Social Class		
ST	2	
OBC	1	
Schooling attained		
11–12th Class	1	
College	2	
Number of PLA sessions attended		
1–3	1	
4–5	1	
>6	1	
**Health workers (n=23)**	**10**	**1 FGD of ASHAs (13 participants)**
ASHA (community health worker)	3	13
ASHA Supervisor (cluster of villages)	1	
Auxiliary Nurse Midwife (village level primary healthcare MCH worker)	3	
Community health doctor (secondary level care)	1	
District hospital adolescent reproductive and sexual Health counsellor (tertiary level care)	1	
District obstetrician (tertiary level care)	1	
**Unstructured PLA group observations**		**5 PLA Groups (≈ 175 participants)**
	**Total number of interviews=29**	**Total number of FGDs=9**, **Total number of participants in FGD=63**, **Total number of PLA groups observed=5**

* Mixed group of pregnant, parenting and unmarried adolescent girls; of the 33 participants, 14 were pregnant.

ASHA, Accredited Social Health Activist; FGD, focus group discussion; MCH, maternal and child health; OBC, Other Backward Class; PLA, participatory learning and action; SC, Scheduled Caste; ST, Scheduled Tribe.

Data collection spanned different agricultural seasons (from December 2018 to December 2019) and ended before the COVID-19 pandemic. Interviews and FGDs were audio recorded and conducted privately in Hindi by two Ekjut researchers (SR, SDT), with FS present for most data collection. The district doctor was interviewed in English by FS. One Ekjut researcher (SR) has extensive qualitative interviewing experience in Jharkhand and trained the second researcher (SDT).

We used several techniques to engage shy adolescent girls. A safe and private space was selected for data collection; this was the girls’ home, if appropriate, or otherwise a community space, such as the local health centre. Another member of the research team simultaneously interviewed the family to prevent adults from eavesdropping. All researchers were women (which was culturally necessary) and in their 30s. No researcher originated from the villages where data were collected, but the two main interviewers were Indian and lived in Jharkhand. The youngest interviewer was unmarried (signalled through lack of public marriage symbols, for example, *mangalsutra*), which may have affected her perceived authority to ask questions about marriage and pregnancy.

One issue was clarifying participants’ age and hence determining if they were adolescents. Some girls and health workers inflated pregnant adolescents’ ages, due to fears of ineligibility for health cash incentives and concerns around reporting early marriage. All pregnant adolescents were married, including those who became pregnant before marriage. No participant said they knew unmarried pregnant girls. After initial rapport building and reiterating interview confidentiality, we asked about the timing of the highest education level attained to clarify age. We did not probe age during FGDs. Three adolescents interviewed were pregnant for the second time: one had lost their firstborn, another miscarried and one had a surviving child.

Data were translated into English by a bilingual speaker. Some participants occasionally used local languages, but most conversations were in Hindi. To ensure accurate translation, all translated transcripts were reviewed line by line by the respective interviewer (SDT, SR).

### Data analysis

We wanted to give voice to the experiences of pregnant adolescents while acknowledging the many restrictions that constrain their ability to express themselves. This required a flexible approach. We chose inductive thematic analysis as described by Braun and Clarke,^24^ using an approach drawing on critical realism^25^ as well as Swartz’s navigational capacity framework^26^ to inform inductions. Critical realism combines realist ontology with a constructivist epistemology, thus helping to validate girls’ perspectives as reality and capturing latent themes that might not have been directly expressed. We used Swartz’s navigational capacities framework to inform our exploration of latent themes. Developed for youth in the Global South, the framework recognises that youth face many structural constraints that can thwart their development and outlines a broad range of individual and collective capacities needed for them to flourish.

Two researchers (SDT, FS) read through all transcripts to familiarise themselves with the data. Initial impressions were shared between three researchers (FS, SDT, AP). SDT cross-checked linguistic nuances and cultural references with local PLA group coordinators. FS conducted the first round of open coding in Nvivo (V.1.5.1) using semantic methods. The codes were then reviewed together by two researchers (FS, SDT) and a list of initial themes generated. Another round of coding was done, this time inductively and drawing on theories from the literature as well as the initial semantic coding. Three researchers (FS, SDT, AP) discussed the generated themes together. FS then conducted a third round of coding using the coding framework developed and checked that the final set of themes was salient both within and across transcripts. These generated themes were further discussed through joint consultations between FS, AP, GP and SDT.

The navigational capacities framework was used to further interpret and consolidate codes and informed further thematic articulation. The final themes were reviewed and discussed with all researchers (FS, SDT, SR, NN and AP) spanning multiple disciplines and including researchers based in Jharkhand (SR, NN, SDT). Jharkhand-based researchers led the study design, data collection and collaborated on theme generation. The final check of themes was done by local senior researchers. More details on author reflexivity can be found in the online supplemental file 1. Findings are reported according to the Consolidated Criteria for Reporting Qualitative Research guidelines (see online supplemental file 1).^27^

### Role of the funding source

The funder of the study had no role in the study design, data collection, data analysis, data interpretation or writing of the report.

## Results

We found that overall pregnant adolescents’ engagement with PLA groups was limited. Only one of the 15 pregnant adolescents in our study who had attended groups had attended more than six meetings, and 10 had attended between one and three meetings. This was partially due to girls being new to their marital village and not connected to local services, but also due to the many restrictions pregnant adolescents faced, which impacted their ability to leave the house and attend social gatherings.

**Figure 1:**
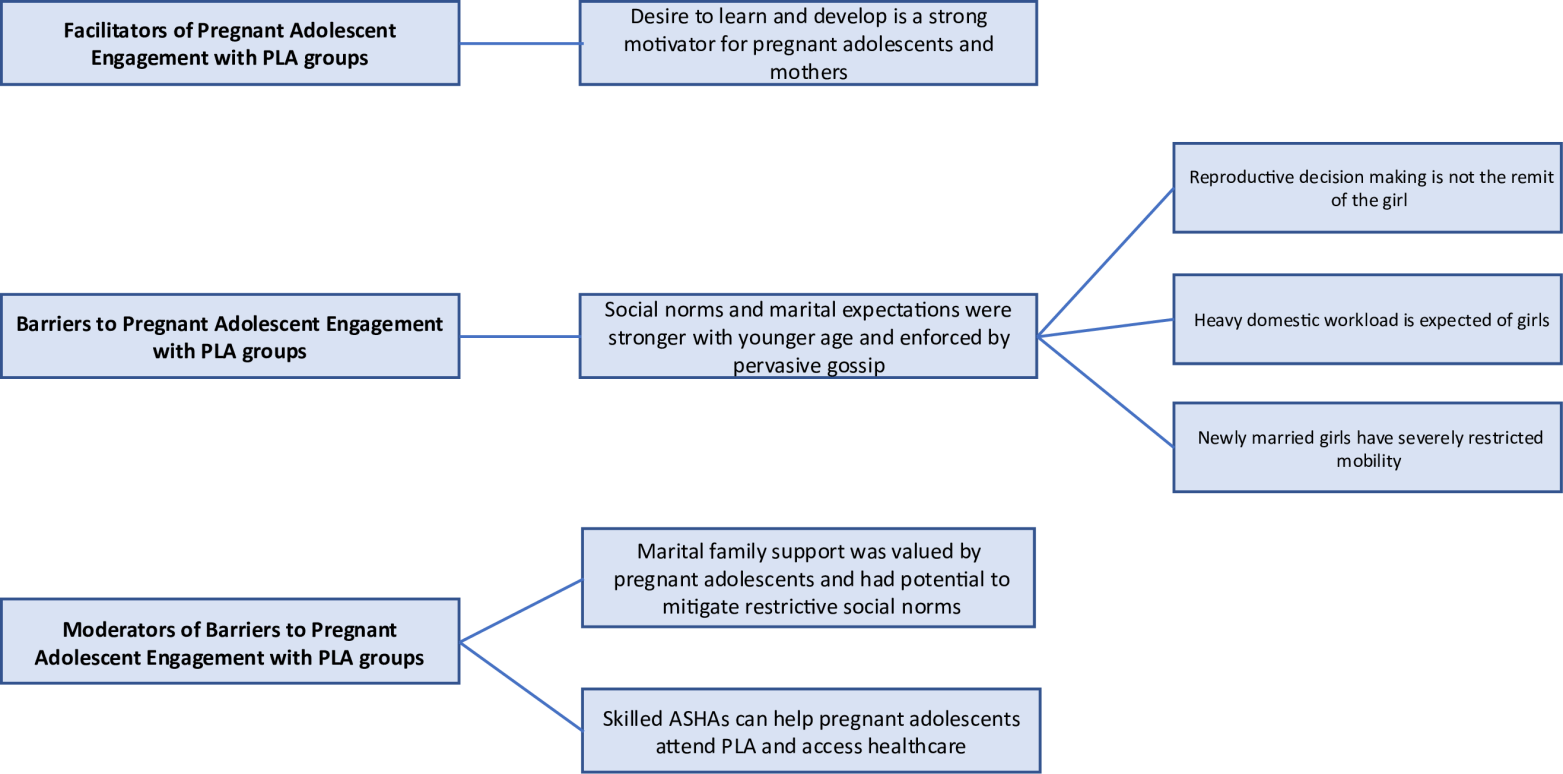
Pregnant adolescents’ engagement with PLA groups identified themes. ASHA, Accredited Social Health Activist; PLA, participatory learning and action.

**Figure 2:**
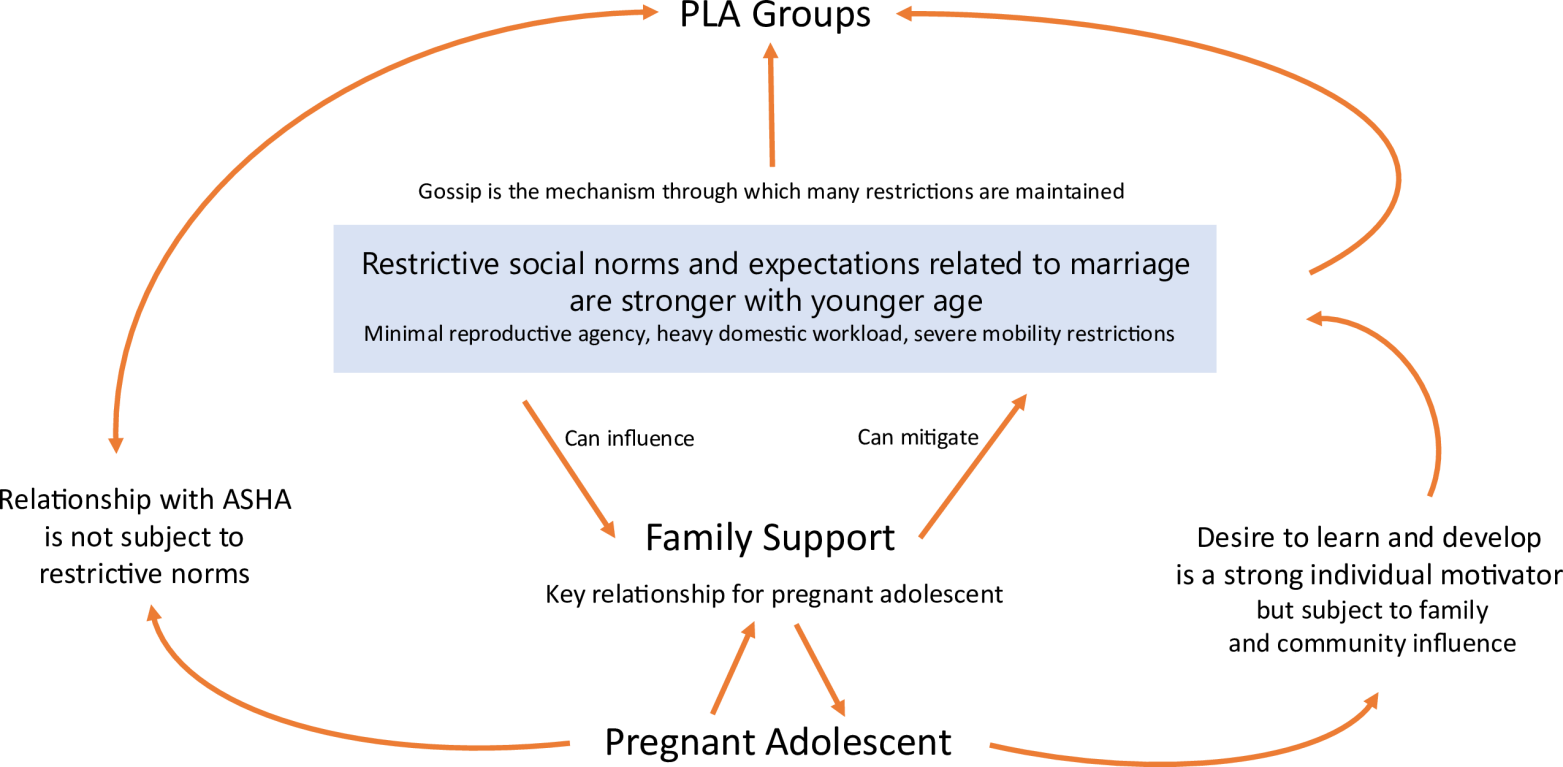
Diagram of interactions between identified themes influencing pregnant adolescents’ engagement with PLA groups. ASHA, Accredited Social Health Activist; PLA, participatory learning and action.

We identified a number of themes that captured pregnant adolescents’ engagement with PLA groups (see figure 1). A *desire to learn* facilitated engagement with groups, but *restrictive marital norms that were stronger with younger age and enforced by pervasive gossip* impeded it. Two main factors potentially moderated the negative influence of restrictive marital norms to enhance engagement: *marital family support* and *a skilled ASHA* (see figure 2).

### Facilitators of pregnant adolescents’ engagement with PLA groups

#### Desire to learn and develop is a strong individual motivator

All pregnant girls were keen to learn. They enjoyed attending PLA groups and wanted to be in a space where they could learn from others.

How will one get this knowledge if [one] doesn’t come to the meetings? Everyone has their own knowledge but when you come to the meetings, talk to people, discuss, then your knowledge increases. (≤19-year-old OBC primigravida who attends PLA groups)

The content of PLA meetings was particularly resonant for pregnant adolescents.

Interviewer: Okay…. what is good about it [PLA groups]?

Respondent 2: The difficulties in becoming a mother… [they talk] about it. Is there a facility to go to the hospital from the village or not? I liked that too. (mixed FGD B)

The opportunity to come together in a social space and hear from others was also valued.

Interviewer: So you go because you get to learn… What else do you like there?

Respondent: I enjoy being with everyone there… Discussion about everything takes place… this and that….This does not happen at home. (≤ 19-year-old OBC primigravida who attends PLA groups)

Most girls felt that groups had a positive effect in the village and improved behaviours such as handwashing and seeking timely medical care. Yet, having a strong personal desire to learn alone was insufficient for participation in PLA groups, as girls had to overcome many restrictive norms.

### Barriers to pregnant adolescents’ engagement with PLA groups

#### Social norms and marital expectations were stronger with younger age and enforced by pervasive gossip

Being an adolescent girl in a rural village brought behavioural obligations and expectations, as highlighted by participants: “This is a village not a city. You can’t live on your own terms here” (FGD B - adolescent girls). Key social norms we identified included restricted reproductive agency, severe mobility restrictions and heavy domestic workload expectations. These restrictive norms greatly impacted how pregnant adolescents could engage with PLA groups. In general, these social norms were more restrictive the younger the pregnant adolescent was and the more recently she had been married. This hindered PLA group participation more for pregnant adolescents than their adult or unmarried adolescent counterparts.

95% do come but about 5% are still in the grip of their mother-in-law. The ones who are recently wed they are the ones dependent on their mother-in-law and husband. (ASHA FGD)

Many capacities developed through PLA cycles, such as expressing and prioritising common problems in pregnancy, were not seen as desired capabilities for married girls, with adolescents expressing that newly married girls had no voice in their marital homes.

[…] after marriage life changes and you cannot voice your opinions at your in-laws’ place.… After marriage you are treated like a machine, go there, and do this, and we have no opinion, we just follow. In our parental home, they also decide for us, like arrange our marriage, but at least when we voice our opinion, they listen to us… At least someone considers our opinion. (mixed FGD A)

Pregnant adolescents wished to make reproductive choices but perceived this as impossible.

… we go to our in-laws place after marriage, we are not given the chance to think when we want to be a mother and when not to be a mother… (mixed FGD B)

This constraint on expressing one’s opinions likely influenced participation in PLA groups. No pregnant adolescent we interviewed had ever spoken at a PLA group session. However, some expressed being overwhelmed by pregnancy, not even knowing what questions one should ask to begin with.

Interviewer: Did *Aanganwadi* Didi (maternal and child health worker) give you the expected delivery date?

Respondent: No. I don’t know anything. I am new. I don’t know what to do and how things are to be done… I don’t know that we have to ask them. (≤19-year-old ST primigravida living in an area without PLA)

Despite some exceptions, newly married girls were often severely isolated. They were not permitted to leave their homes unless accompanied by a guardian, and when no family member was home, only the ASHA could legitimately accompany them to health services. Heavy domestic workloads, particularly from poorer girls with farming obligations, also restricted ability to attend groups.

Why I don't come (to PLA groups) is because I stay alone in the house. No one is there. So, I have to do all the housework… cleaning and cooking. So, do not get the time sometimes. (mixed FGD B)

Gossip was a mechanism often used to reinforce restrictive social norms. Unsupportive families would share negative gossip about daughters-in-law as a form of social control enforced through community scrutiny. Unsupported married girls described the pressure of scrutiny and criticism as a source of great distress.

In your marital home everyone is commenting on you but before marriage only our parents correct us. Say what you want to my face! (referring to community gossip) (mixed FGD C, with unmarried and pregnant girls)

Gossip was so pervasive that even if families did not enforce some restrictions, girls self-imposed them to avoid attracting undue gossip and its negative consequences. Some girls, for example, chose not to leave their homes, as they knew doing so could trigger gossip, even if they were advised by the ASHA to seek others’ help for pregnancy support. Village gossip had less impact on girls who had supportive marital families, particularly if they continued their education and were less confined to the village.

Families appeared to have different expectations of adult primigravidae compared with pregnant adolescents. For example, pregnant adults had greater mobility and flexibility to attend community activities, had greater say in their marital families and less domestic work expectations.

Interviewer: How do they help you?

Respondent: If I do not want to cook food or could not cook, then they did it. If I could not do any work they did it.

Interviewer: Your mother-in-law cooked food for you?

Respondent: Yes.

(>20-year-old adult OBC primigravida who attends PLA)

### Moderators of barriers to pregnant adolescent engagement with groups

#### Marital family support was valued by pregnant adolescents and had potential to mitigate restrictive social norms

Although marital families could impose stifling restrictions on pregnant adolescents, girls still wanted and greatly valued their support. In our observations of PLA groups, we found that ASHAs spent most of the meeting sharing information, and although there was space for participation, this would be dominated by older women. Despite this, all married girls expressed a preference to have in-laws and elders present in PLA groups and also implied that elder approval would be needed to implement any health behaviour changes.

Interviewer: These meetings that you attend. There are old women, women of your age, women the age of your mother and even small girls. Should the meeting of all be conducted separately or together?

Respondent: Everyone should be there.

Interviewer: Why?

Respondent: As the old will teach the young, their daughter and daughter-in-law, what they should do. And the young will get to know things.

Interviewer: If they will not be there and only your age girls are there? Do you not feel that if they are there you are not able to speak? Is there no such thing?

Respondent: No. It should be, we should speak.

(≤19-year-old ST secundigravida who attends PLA-C)

Another young mother described the benefit of having mothers-in-law reinforce and explain what she learnt in groups, “I may not be able to understand but they (mothers-in-law) do. They may explain it to me and I may understand” (≤19-year-old OBC woman who attends PLA groups). Girls wished to have collective support from their elders as they navigated the new stage of pregnancy. Even a girl who had opted for a ‘love marriage’ and so had lost family support bemoaned not having a mother-in-law’s advice during pregnancy.

Yes, I think if my mother-in-law was around and could tell me things that would be good… (≤19-year-old ST primigravida)

Due to the restricted mobility placed on married girls, they had limited or non-existent social networks, and thus relied on their marital family for health information. Many girls relayed that they turned to their husbands with questions about their pregnancy. None of the men we spoke to attended PLA groups or appeared well informed about pregnancy. The role of men in reproductive decision-making extended beyond advice; critical health decisions, such as opting for institutional delivery, often required funds which were controlled by fathers-in-law. A supportive and well-informed family was thus in many ways critical for the health and even survival of pregnant adolescents.

In addition to health, girls conveyed that it was their marital families, including their husbands, who largely determined the opportunities open to them after marriage. Marital families could decide to break social norms, such as overcoming restricted mobility to continue schooling. A supportive marital family was seen as critical to achieving one’s aspirations for both married and unmarried girls.

Some husbands permit it (education), some don’t. If the father-in-law and mother-in-law are good, they will let her study if she wants to. But if the husband is not good, he will make her work in the fields. That is what one has to do after marriage. (FGD with family of ≤19-year-old OBC mother who attends PLA)

Pregnant adolescents lived with their marital families who ranged from being supportive to restrictive. A family could strongly reinforce negative social norms, isolate a girl socially and prevent her from attending PLA groups. These restrictions could be abusive, leading to poor outcomes for mother and child. One pregnant adolescent described being subject to severe restrictions including being prevented from attending PLA groups for fear that engaging with others would make her less obedient at home, “…it was thought that the other women misled me to fight.” She described the heavy domestic work required of her when pregnant, which led to illness, weight loss and the tragic subsequent death of her newborn.

My marital home is near a jungle…. I lived with my aunt-in-law. She used to send me to the jungle (when pregnant). We lose weight when we have to cut wood. Then to draw water from the well. (≤19-year-old ST mother who experienced the death of first child as a neonate)

Other in-laws were more ambivalent about the benefits of PLA groups, as they had managed their own pregnancies without them, but with some encouragement from the ASHA, would send their daughter-in-law to groups. More supportive families assisted the girl with domestic work, encouraged her to continue studying and attend PLA groups. The most supportive families were those that prevented early marriage and adolescent pregnancy to begin with. One indigenous adult primigravida who lived in a village alongside those experiencing restrictive conditions described her father encouraging her to continue her studies and delay marriage, “My father is not actually literate, but he thought the best for me.” Within her marital home, she was well supported.

Interviewer: When you were pregnant did your family here take care of you?

Respondent: My husband and my elder sister in-law. She even served me food on my bed. In the beginning I could walk around but in the final months all were helpful. In last month everyone took great care of me… including my husband, sister in-law… everyone…” (>20-year-old adult ST mother of first child)

Ultimately, how pregnant adolescents engaged with PLA groups and the extent to which they could implement what they learnt in them was highly dependent on their marital family’s emotional, physical and financial support. Such huge diversity in family support was seen across girls from the same caste or tribal group. Pregnant adolescents from more restrictive families were at risk of poorer outcomes and in such cases often the only legitimate avenue of support for these girls was the ASHA.

##### Skilled ASHAs could help pregnant adolescents attend PLA and access healthcare

ASHAs could often legitimately bypass restrictions placed on pregnant adolescents. A capable and well-respected ASHA could accompany a young married girl out of the house to PLA groups and to clinic or hospital appointments. Almost all pregnant girls mentioned that they would first turn to the ASHA for help with their pregnancies (online supplemental table 1). Health professionals also relied on ASHAs for blood tests, antenatal records and to reinforce their advice. Many used various techniques to specifically engage adolescents, such as talking to them privately and in regional languages (online supplemental table 1).

ASHAs also faced challenges in reaching pregnant adolescents. Several interviewees mentioned that ASHAs preferred to call educated women to groups as they were better able to participate. ASHAs also tended to invite those who lived closer to them because they were easier to visit. Not all girls praised the ASHA, noting that some requested money or were uninterested in helping. Overall, however, ASHAs were supportive and many requested more training to better engage adolescents. Engagement with adolescent males was another skill ASHAs wished to develop, though they acknowledged this would be difficult.

## Discussion

This is one of the few studies from a low- and middle-income country to explore pregnant adolescents’ engagement with a perinatal health intervention from their own perspectives, and the only study to explore pregnant adolescents’ engagement with women’s groups practising PLA. All girls had a desire to learn and improve their lives, and all wished to attend PLA groups and meet others. However, girls also recognised the need to play by ‘village rules.’ Pregnancy was intimately tied to norms related to marriage which were stronger for younger girls. These norms and expectations restricted girls’ reproductive agency and mobility, demanded heavy domestic workloads and had substantial implications for their engagement with PLA groups. There was, however, great diversity in how these norms were applied across marital families, with supportive families mitigating restrictions and enabling pregnant adolescents to attend PLA groups. Pregnant adolescents wished for and valued having supportive marital families, and all expressed wanting their elders present in PLA groups to learn from them. The role of the marital family in a pregnant adolescent’s life and well-being was critical. Pregnant adolescents were lost, alone and often confused, and although most did not desire marriage and pregnancy, they understood that, in those circumstances, a supportive mother-in-law and husband were crucial. This support had the potential to extend to support for their aspirations such as continuing with schooling and to feel loved and respected rather than scrutinised and criticised through gossip. Girls described the desire to have their in-laws be like loving parental figures who could guide them through their pregnancies and also in their lives. Our study found an important role for marital families, in particular in-laws, in the ongoing development of pregnant adolescents with the potential to view them as enablers and parental figures that could enhance engagement of pregnant adolescents with PLA groups as well as broader adolescent developmental outcomes.

Our findings challenge some of the assumptions about the role of in-laws in pregnant girls’ lives. North and Eastern India, including Jharkhand, has been described as having strong and deeply entrenched patriarchal norms.^28 29^ These norms include a patrilocal system that encourages early marriage and fertility within traditional gender roles where the daughter-in-law is the most subordinate family member.^30^ These experiences are not limited to India. In Burkina Faso, for example, pregnant married girls have limited decision-making capacity, and domestic duties are expected to be prioritised above healthcare.^31^ In such settings, marital families are primarily viewed negatively, as enforcers of restrictive social norms; interventions then often focus on enhancing girls’ reproductive decision-making by reducing marital family interference or engaging marital families to make them more supportive of the girl, but do this without adolescent-related developmental considerations, such as the need for parental guidance and the value of in-laws in the girl’s own development.^32^,^34^ Our findings show a much more dynamic interplay between social norms and support within a girl’s marital family, with some families offering strong, enabling support and others being more restrictive. Given the crucial role of parenting in adolescent development generally,^35^,^37^ it is not surprising that married girls in Jharkhand who face the added stressors of pregnancy and motherhood wanted supportive parent-like involvement from their marital family.

Our results raise several important issues for future programming to support pregnant adolescents. Although evidence is limited, engaging parents as a source of support for adolescents during pregnancy and motherhood may lead to better outcomes. In a doula training programme in Chile, most adolescents chose their own mothers to be their doula, which related to more positive pregnancy experiences.^6^ In Thailand, mothers supporting their adolescent daughters to breastfeed led to higher exclusive breastfeeding rates at 6 months,^38^ with similar findings in Brazil.^39^ In Jharkhand, even though girls were married and faced multiple restrictions, they still expressed wanting supportive families, but expressed this as having supportive in-laws, the only accessible parent-like figures to help and support them during their pregnancies.

Research and programmes to support pregnant girls would benefit from a framework that both acknowledges the stifling restrictions of early marriage but also works constructively with these restrictions to support the development of pregnant adolescents and their children. Swartz’s conceptual framework for navigational capacities,^26^ which draws on Appadurai’s work,^40^ values agency but also recognises the many other individual and collective navigational capacities that youth in low-resource settings require to deal with the structural constraints they face. Frameworks that originate in the Global North, such as resiliency theory, can potentially result in individuals blaming themselves for what are instead structural failures, bringing despondency and hopelessness.^26^ The girls we interviewed displayed many unrecognised navigational capacities, such as being able to understand the rules that governed their environment and a strong desire to learn. Community interventions such as PLA groups, where everyone comes together to plan, act and reflect and build collective agency, can potentially play a larger developmental role for girls by helping to nurture navigational capacities that will help them better navigate the constraints they face.

For girls in the most stifling family environments, however, effective ASHAs are needed to enable girls to even attend PLA groups. Other studies from low- and middle-income countries have suggested that with some adolescent-friendly training, local community health workers can build rapport and trust with pregnant adolescents and connect them with services.^41^,^43^ Our study mirrors these findings and reinforces the need to consider additional training and support for ASHAs so that they can meaningfully engage with pregnant adolescents and their families and link them to programmes and services. ASHAs also have a role in engaging men, who in our study were an untapped source of power and support for pregnant adolescents. Consistent with increasing recognition of the value of engaging men in maternal and reproductive health,^44^ we found that husbands often made significant reproductive health decisions. Strengthening India’s flagship adolescent community health programme, *Rashtriya Kishor Swasthya Karyakram*, so that it reaches all villages^45^ is one approach to enhance reproductive health knowledge in males and females well before marriage.

Many of the limitations of our study apply to any qualitative research with pregnant adolescents. Such studies will almost always be conducted by those with a worldview that values formal education with its implicit requirement to delay pregnancy. For example, two of the researchers involved in data collection were medical professionals trained to counsel adolescents to avoid early pregnancy. This creates a ‘disconnect’ in worldviews and power differentials in data collection. We strove to be conscious of these effects and to recognise our privilege while interacting with pregnant adolescents. In our study, differences in experiences and worldviews were compounded by the influence of rurality and indigenous cultures, with differences in language and conceptions about time and preventative health. Although all participants spoke Hindi, they would occasionally turn to tribal languages, indicating their preference. Some of our questions on future planning and aspirations were often met with puzzled questioning about the usefulness of preparing for chance occurrences. While these chasms in worldviews need acknowledging, they do not minimise the importance of studying pregnant adolescents’ perspectives, whose views will otherwise continue to be ignored in programming. The collaboration with Ekjut was fundamental to access this population. However, as proponents and trainers of PLA, Ekjut staff had strong convictions about its benefits. This influenced both data collection and interpretation, but was countered by researchers not affiliated with Ekjut, who observed data collection, debriefings and conducted data analysis.

## Conclusion

Pregnant adolescents’ experiences and engagement with PLA groups in rural Eastern India are diverse. Those who were able to attend PLA groups thought that they were informative, helpful and provided space for much wanted social engagement. Although many pregnant adolescents faced multiple and severe restrictions that impacted their ability to engage with PLA groups, these restrictions were dynamically moderated by marital family support. Maximising pregnant adolescents’ engagement with PLA groups would benefit from greater engagement with marital families as key influences on girls’ well-being and development. Very restrictive families require more intense encouragement, follow-up and effort from ASHAs.

These findings have broader implications for supportive programming for married girls. Girls thrust prematurely into marriage and motherhood are still adolescents with fundamental developmental needs, including for supportive families that do not reinforce restrictive norms. Recognition of these developmental needs can reframe approaches to supportive programming in the context of early marriage, including viewing the role of the marital family as important in nurturing girls’ own capacities.

## Supplementary material

10.1136/bmjph-2024-001309 online supplemental file 1

## Data Availability

Data are available upon reasonable request.
