# Supplementary material for: Pregnant adolescents’ dynamic engagement with participatory women’s groups for maternal and newborn health in rural India: a qualitative study
Source: BMJ Public Health. 2025 Aug 14;3(2):e001309. doi: 10.1136/bmjph-2024-001309 (PMC12352144; doi:10.1136/bmjph-2024-001309)
Supplement: online supplemental file 1 [file bmjph-3-2-s001.docx]

# Supplementary File

# Contents Page

1. Distress Protocol 1
2. Consolidated Criteria for Reporting Qualitative Studies 2
3. Table 1 - Representative Quotes 5
4. Sample interview guide 21
5. Author Reflexivity Statement 25

# Distress Protocol

## ***Project:*** Exploring Adolescent Pregnant Girls engagement and empowerment through participatory women’s groups

Discuss with Local Community Health Worker (ASHA) regarding suitability of participant for an interview or participation in focus group discussion. Note Exclusion Criteria of: recent perinatal loss, facing severe family violence or abuse, severe malnutrition or severe depression, or if mother or her infant is acutely unwell.

If there is still psychological distress expressed or detected in the interview or disclosure of family violence or other victimisation the following steps should be taken:

Psychological Distress during interview expressed or detected

Disclosure of Family Violence or other Victimisation

Offer confidential referral for assessment and counselling by Ekjut Counsellor

Give phone numbers for local police (district women’s police officer, child protection officer)

Discuss case with Dr Nirmala Nair, in order to identify appropriate referrals to local health, and legal services

If ongoing distress or need for further evaluation, referral to local health services

# Consolidated criteria for reporting qualitative studies (COREQ): 32-item checklist

| **No. Item** | **Guide questions/description** | **Reported on Page #** |
| --- | --- | --- |
| **Domain 1: Research team and reﬂexivity** |  |  |
| *Personal Characteristics* |  |  |
| 1. Interviewer/facilitator | Which author/s conducted the interview or focus group? | 6 |
| 2. Credentials | What were the researcher’s credentials? E.g. PhD, MD | 1, 14 |
| 3. Occupation | What was their occupation at the time of the study? | 6, 7, 14 |
| 4. Gender | Was the researcher male or female? | 6 |
| 5. Experience and training | What experience or training did the researcher have? | 6 |
| *Relationship with participants* |  |  |
| 6. Relationship established | Was a relationship established prior to study commencement? | 5, 6 |
| 7. Participant knowledge of the interviewer | What did the participants know about the researcher? e.g. personal goals, reasons for doing the research | 5 |
| 8. Interviewer characteristics | What characteristics were reported about the interviewer/facilitator? e.g. Bias, assumptions, reasons and interests in the research topic | 6, 14 |

| **Domain 2: study design** |  |  |
| --- | --- | --- |
| *Theoretical framework* |  |  |
| 9. Methodological orientation and Theory | What methodological orientation was stated to underpin the study? e.g. grounded theory, discourse analysis, ethnography, phenomenology, content analysis | 7 |
| *Participant selection* |  |  |
| 10. Sampling | How were participants selected? e.g. purposive, convenience, consecutive, snowball | 6 |
| 11. Method of approach | How were participants approached? e.g. face-to-face, telephone, mail, email | 6 |
| 12. Sample size | How many participants were in the study? | 6 |
| 13. Non-participation | How many people refused to participate or dropped out? Reasons? | 6 |
| *Setting* |  |  |
| 14. Setting of data collection | Where was the data collected? e.g. home, clinic, workplace | 6 |
| 15. Presence of non-participants | Was anyone else present besides the participants and researchers? | 6 |
| 16. Description of sample | What are the important characteristics of the sample? e.g. demographic data, date | Table 1 |
| *Data collection* |  |  |
| 17. Interview guide | Were questions, prompts, guides provided by the authors? Was it pilot tested? | 5,  Appendix p.21 |
| 18. Repeat interviews | Were repeat interviews carried out? If yes, how many? | NA |
| 19. Audio/visual recording | Did the research use audio or visual recording to collect the data? | 6 |
| 20. Field notes | Were ﬁeld notes made during and/or after the interview or focus group? | 6 |
| 21. Duration | What was the duration of the interviews or focus group? | Appendix. p.21 |
| 22. Data saturation | Was data saturation discussed? | NA |
| 23. Transcripts returned | Were transcripts returned to participants for comment and/or correction? | NA |
| **Domain 3: analysis and ﬁndings** |  |  |
| *Data analysis* |  |  |
| 24. Number of data coders | How many data coders coded the data? | 7 |
| 25. Description of the coding tree | Did authors provide a description of the coding tree? | Figure 1, 7 |
| 26. Derivation of themes | Were themes identiﬁed in advance or derived from the data? | 7 |
| 27. Software | What software, if applicable, was used to manage the data? | 7 |
| 28. Participant checking | Did participants provide feedback on the ﬁndings? | 5 |
| *Reporting* |  |  |
| 29. Quotations presented | Were participant quotations presented to illustrate the themes/ﬁndings? Was each quotation identiﬁed? e.g. participant number | pp.8-12 |
| 30. Data and ﬁndings consistent | Was there consistency between the data presented and the ﬁndings? | pp 8-12 |
| 31. Clarity of major themes | Were major themes clearly presented in the ﬁndings? | pp8-12 |
| 32. Clarity of minor themes | Is there a description of diverse cases or discussion of minor themes? | Pp8-12 |

Developed from: Tong A, Sainsbury P, Craig J. Consolidated criteria for reporting qualitative research (COREQ): a 32-item checklist for interviews and focus groups. *International Journal for Quality in Health Care*. 2007. Volume 19, Number 6: pp. 349 – 357

| **Table 1: Representative Quotes** | |
| --- | --- |
| **Marital social norms and expectations are stronger with younger age** | ***Early marriage and pregnancy is expected norm***  after marriage life changes and you cannot voice your opinions at your in-laws place. If you want to study and have food you cannot voice your own opinion. After marriage you are treated like a machine, go there and do this, and we have no opinion, we just follow. In our parental home they also decide for us, like arrange our marriage, but at least when we voice our opinion they listen to us…at least someone considers our opinion. (mixed FGD A)  Respondent 4: 95 % do come but about 5% are still the grip of their mother-in-law. The ones who are recently wed they are the ones dependent on their mother-in-law and husband. (ASHA FGD)  Interviewer: When someone gets pregnant very early how do the villagers look at her? Do they help her? We all know the age is 18 years but if someone does get pregnant early than what happens?  Respondent 4: The villagers are happy. They think that she came and got pregnant.  Respondent 7: The villagers think that it is very good. She came and got pregnant. Her womb is fertile. (ASHA FGD)  ***A village has its own rules***  Village is not a city – it has different rules… We are not able to send a girl away from home for further training. I had the opportunity to go for further training, but the villagers refused and then one of them spoke to my father… (mixed FGD A)  ***Discordance between social norms and personal beliefs***  Respondent: I did not want to get married at this age still I got married. I wanted to study but just could not as I was married. What could I do?  Interviewer: You did not tell your family that you wanted to marry after completing your education?  Respondent: No, I did not say anything.  Interviewer: Why?  Respondent: Just like that. (16-year-old SC primigravida living in control area 3)  Interviewer: Would you prefer a baby when you were 17-18 or at the age you are now?  Respondent: I am happy now.  Interviewer 2: Why?  Respondent: No one wants to have a baby at that age. (23-year-old OBC adult primigravida B attends PLA)  ***Aspirations for own daughters differ***  Respondent: Whatever has happened to me (early marriage), it has happened. But I won’t repeat that with my children. (18-year-old OBC primigravida attends PLA - A)  ***Exceptions to norms***  Interviewer 2: If I understood properly your father did not allow you to get married early. How is your father’s thinking like this?  Respondent: My father is not actually literate but he thought the best for me.  Interviewer 2: did the villagers pressurise your father?  Respondent: No, never. My father does not listen to the villagers, he does what he wants. What he thinks is right.  (24-year-old ST primigravida A attends PLA) |
| **Marriage is between families and not individuals, and its purpose is to produce children** | I felt good (when I became pregnant). What will I do? See once you get married you need to have kids. If you do not have kids then you will have to listen to abusive words.  (18-year-old ST secundigravida, mother of 1.5-year-old, no PLA)  Here, things don't work according to our wishes. It works according to our family members’ wishes. Means as per Mother-in-law and father-in-law.  (mixed B FGD)  She should also have early pregnancy so that she can prove her fertility. Also, they favour normal delivery and do not want to understand high risk factors. We have to explain it to them. Some agree with us whereas others don’t.  (Community health centre doctor)  Respondent:…... I got married as per my parents wish….  Respondent: I was in 9th class when I was getting married.  Interviewer: You were in 9th class?  Respondent: I did not want to get married but my parents wanted me too. I was refusing but it was not cancelled. Once it got cancelled but the second time I was married.  (19-year-old ST secundigravida attends PLA - C) |
| **Reproductive decision making is not the remit of the girl** | Respondent: I had told myself and also to my husband that we will not have children. My husband also said so, but after a year my mother-in-law started to say that it has been so many days and the ones who were behind me had done then why not she.  (19-year-old OBC primigravida no PLA 2)  Respondent: the decisions for my first pregnancy were made by my mother-in-law….and now I know for my second pregnancy that I have to take the decisions about what I must do (adolescent girl who lost first neonate)  (mixed FGD A)  Interviewer: Who decides for the pregnant women in your village? Can a woman decide whether she wants to go or not?  Respondent 4: No. In the village mostly the mother-in-law or husbands decides for them. If ones husbands is good than she can go anywhere with his decision but if ones husbands is more towards his mother than she can go after they both allow her to. Here you will hardly find one who can decide for oneself. Some even accuse us that we are telling them something which will create dispute in the family.. they have fear of losing their power over their own daughter in law…  (ASHA FGD)  When we tell the women directly they will not come for PLA meetings but if we tell their mother in law or their husbands then they do send them or accompany them. If we will only talk to the pregnant woman she will not be able to come.  (ASHA FGD)  Respondent 3: I think that when we go to our in-laws place after marriage, we are not given the chance to think when we want to be a mother and when not to be a mother. The grandmothers want to see the face of their grandchild at the earliest.  …..Respondent 3: And whenever we say that we are not ready for it, we do not want to be a mother now, we want to take care of our needs and take care of our health as the baby can live healthily, right, moving forward?  …Respondent 3: So this is one wrong thinking of theirs that they will be happy when they see their grandchildren. …..They just care that all their needs of drinking and smoking be fulfilled. When a child comes, no one thinks about the future of their life.  (mixed FGD B)  Interviewer: Means who decides what is to be done and what is not to be done? You decide by yourself or the family members decide?  Respondent: Family members decide.  Respondent: Here, things don't work according to our wishes. It works according to our family members’ wishes. Means as per Mother-in-law and father-in-law.  (mixed FGD B)  Respondent: Means if it happened because of our own will (become pregnant), it would have been nice. I want to have a child after so many days and not now. They don't give us the chance to think about it.  (mixed FGD B)  They just want a baby in the house soon, and so, we have to oblige quickly. And then, we face problems.  (mixed FGD B)  Interviewer: The girl can’t decide independently whether she wants to keep the child or not.  Respondent: Yes, it is decided after everyone’s opinion. They do ask their guardian.  (ASHA 3)  ***Men are major reproductive decision makers even when uninformed***  Interviewer: Who helped you at home?  Respondent: My husband and no one else……  Interviewer: What did he tell you?...  Respondent: He told me not to eat stale food and to get up early in the morning. He told me all this.  Interviewer: Who told him all this?  Respondent: I don’t know. He might have taken help from someone. He might have asked someone. He told me all this.  (16-year-old SC primigravida in control area A)  ***Discordant quotes***  Respondent: No. I do not want to take them. We will get operated. Whether I have a boy or a girl.  Interviewer: You decided this or your husband?  Respondent: We decided this together.  Interviewer: You decided that you do not want more children.  Respondent: Yes  (18-year-old ST secundigravida never attended PLA) |
| **Heavy domestic workload is expected of girls which impacts health and education** | They make them do a lot of work. The ones that are mature do not force them to do heavy work but the others do.  (Subcentre Auxiliary Nurse Midwife 3)  Why I don't come (to PLA groups) is because I stay alone in the house. No one is there. So, I have to do all the housework... cleaning and cooking. So, do not get the time sometimes.  (Mixed FGD B)  I want to study but will I study or take care of my home?  Interviewer: What all do you do?  Respondent: cook food, mopping, wash dishes.  (15-year-old ST primigravida living in control area)  Respondent 1: In your marital home everyone is commenting on you (about your domestic work) but before marriage only our parents correct us.  Interviewer: Do parents say it for our good or bad?  Respondent: Good. (Collectively)  (Mixed FGD C)  Respondent: In the villages the daughters in law are made to work a lot. They tell us about all the chores their mother-in-law tells them to do.  …: Many of them work in fields and cultivate potato, onion and maize. The women of ‘mahato’ caste even have to carry things to market……  Respondent: OBC. They make them do a lot of work. The ones that are mature do not force them to do heavy work but the others do. The women tell this to us and we explain them not to do so.  (Subcentre Auxiliary Nurse Midwife 3)  A girl needs to go to the farm and also to school. My brother does not work in the farm, and just has to worry about his education.  (Mix FGD A)  ***There is less domestic pressure on adult primigravida***  Interviewer: How do they help you?  Respondent: If I do not want to cook food or could not cook than they did it. If I could not do any work they did it.  Interviewer: Your mother-in-law cooked food for you?  Respondent: Yes.  Interviewer: What else?  Respondent: My husband does most of the work.  Interviewer: What do you mean by work?  Respondent: To carry things… like water, any heavy things  (23-year-old OBC adult primigravida B attends PLA) |
| **Newly married girls have severely restricted mobility** | Respondent: I got married so I have to live here. Had I not been married than I would live comfortably in my maternal home. I would roam around freely.  Interviewer: You are not able to roam around freely?  Respondent: No, I do here also but not as my maternal home. I will have to ask my guardians. Then only I can go. If they do not permit than how I go out?  (18-year-old ST mother of two children no PLA)  Interviewer: Is it fine if they (adolescent girls) go without asking permission?  Respondent: Yes, that is also fine. (Collectively)  Interviewer: Immediately after marriage?  Respondent 3: No, it is not so. They have to ask permission or take someone with them. (Collectively, agreed)  (unmarried adolescent girls FGD B)  Respondent 5: It is a village not a city that both (boys and girls) are allowed to go out.  Respondent 8: It is like if son has gone out he will come back. If a daughter is out, call her and ask when will she be back?  (unmarried adolescent girls FGD B)  Interviewer: If you want to go out than who decides?  Respondent: I don’t go out.  (15-year-old SC primigravida in control area)  There is one in my village…. they said my daughter in law is 3 months pregnant please make her card. I told her that on the third Saturday come, we will make it. The mother-in-law and husband said that she will not go out of the house as things have recently changed and she will not go out. I explained to her that if you don’t want her go out alone then come along with her. If you have any other problem ask her to cover her face and then take her back with you. Her mother-in-law and her husband did come but they did not allow her.  (ASHA FGD)  Interviewer: How many months have you been here? Around 6-7 months. Do you go out? Do you talk to others?  Respondent: No. I do not talk to anyone.  Interviewer: Why?  Respondent: Just like that. I do not talk to anyone.  Interviewer: Why do you not go out?  Respondent: No.  Interviewer: Do you want to go out?  Respondent: No. I like to stay at home.  (pregnant adolescent in control area (4))  Girls are not allowed to go as per their wish. Based on what father and mother, outside people think and understand, we have to abide accordingly.  (Mixed FGD B)  Interviewer: Is there any place in the village where you want to go?  Respondent: No, I do not go to anyone’s house. He (my husband) does not let me go.  (18-year-old OBC mother attends PLA)  I can’t do so as my mother-in-law comments saying that I have gone out to gossip. I do not anywhere and just stay at home.  (15-year-old ST primigravida living in control area)  ***Isolation from restricted mobility prevents friends, support, ability to plan and aspire***  Interviewer: Other than that? We are asking this as we want to know that other than your family are there others who can help you?  Respondent: There is no one madam.  (17-year-old ST secundigravida who lost first pregnancy no PLA 3)  Respondent: I don’t have any friends. How can I go to sit anywhere I have to look after my husband also? I don’t go out quickly often. There is no one to listen to my grief.  (17-year-old ST secundigravida who lost first pregnancy no PLA 3) |
| **Family support is a critical safety net that can overcome vulnerabilities** | If someone's family is good, then she will be taken care of. But if someone's family is not good, then no one will take care of her. Then she has to just live her life. And if her family members are understanding, then her husband also supports her and the husband will always take care of what she wants to eat and doesn't want to it, what she wants to do, and what thing will ease her difficulties.  (mixed FGD B)  Interviewer: What support does a young pregnant girl get from home?  Respondent: Those who are sensible they help in carrying things and going to market if she is unable to.  Interviewer: What about the others?  Respondent: They do not help and she has to do it herself.  (Auxiliary Nurse Midwife 3)  Respondent 1: Some husbands permit it some don’t. If the father-in-law and mother-in-law are good they will let her study if she wants to. But even if the husband is not good he will make her work in the fields. That is what one has to do after marriage.  Interviewer: Is it acceptable for you? Getting married in such a house..  Respondent 1: No  Respondent 3: No  Interviewer: What kind of a house do you want then?  Respondent 3: A house where I can study-  Interviewer: What do you want to study? If for example you get an opportunity, God listens to you, what would you like to study?  Interviewer: What do you want to do with your life.  Respondent 3: I want to become a teacher  (family D FGD)  ***Pregnant girls value having someone around to help them***  Respondent: No when I was 3 months I went there to live with my in laws. It was a city area so I did not lose weight so I went there. When they all went for duties there was no one to take care so I came here.  Interviewer: What do you think, if your in laws were at home it would be good? Should there be elders at home when you are pregnant?  Respondent: They should be there.  Interviewer: What help do you get from them?  Respondent: When they stay with us the help we get is we will do nothing wrong. They can also call for doctor or nurse.  Interviewer: They call?  Respondent: If I have to deliver and I am alone than I do not have the strength.  (19-year-old ST secundigravida attends PLA - C)  ***Marital Family can be a critical support through pregnancy***  Interviewer: You are pregnant. Who all support you?  Respondent: Everyone.  Interviewer: How do they support you?  Respondent: Give good food and help me to stay healthy.  Interviewer: What do you mean by good food?  Respondent: Tea and chapatti, vegetables, rice and dal.  Interviewer: Who tells you all this is healthy to have and how to live?  Respondent: Mother-in-law.  (15-year-old SC primigravida in control area)  ***Poor family support***  I have the worst mother-in-law.” (Mixed FGD A) – this girl lost her first newborn due to incorrect advice from her mother-in-law  ***Promises are broken, after marriage things change***  Before marriage in-laws say we will let you continue your education but after marriage they forget about it (Mixed FGD A)  ***Restrictive husband***  Interviewer: Has it ever happened that you wanted to go to the meeting but could not go? Or someone stopped you?  Respondent: Yes  Interviewer: Why does this happen and who stops you?  Respondent: My husband does not let me go out of the house.  Interviewer: Ok  Respondent: He does not like me talking to others.  (18-year-old OBC mother attends PLA)  ***Husbands can be supportive***  Respondent (husband): For example when she needs to bring water, I go with her.  Interviewer: How far is it from here?  Respondent (husband): About half a kilometre  Interviewer: So you go with her?  Respondent (husband): Yes I go with her, pull out the water, give it to her and take it off her shoulders when we reach home.  one.  Interviewer: Okay. What about her diet and nutrition? Have you started something new since she became pregnant?  Respondent (husband): Yes I have done a lot. First she didn’t eat a lot of fruits but now she does... for example... now she eats coconuts, apples, grapes, etc.  Interviewer: Not the names of the fruits.. but you fed her these things?  Respondent (husband): Yes  Interviewer: And she likes it?  Respondent (husband): Yes she likes it.  (Husband of 17-year-old ST primigravida attends PLA - B)  Respondent: I am alone so who will help me. I do it by myself.  Interviewer: You all live together than who helps you?  Respondent: My husband.  Interviewer: How does he help you?  Respondent: He helps in everything.  Interviewer: Like what?  Respondent: When I say anything, he brings. When I say that I can’t do something, he helps a bit with that also.  (19-year-old OBC primigravida no PLA 2)  Interviewer: What help does your husband give?  Respondent: He helps in everything.  Interviewer: What help does he give? You said that you cook food.  Respondent: I cook food but in sitting and sleeping. he take care of the child, he helps. What else can he do?  Interviewer: He takes care of the older child? He feeds him.  Respondent: Yes. He feeds him and I also do so. We both feed him together.  (18-year-old ST mother of two children no PLA)  ***An extended family member can be supportive***  Respondent: My aunt does help. When I have to take food I ask her to do. When something has to be done they do it.  Interviewer: Like what?  Respondent: When I sit for long it starts hurting. So I ask them to do the dishes, they wash them. Anything that has to be done they do like starting the stove. (Wood is used for cooking)  (19-year-old OBC primigravida no PLA 2)  ***Family support for attendance to PLA meetings is critical, and most girls need to be accompanied in order to attend***  Respondent : I don’t go with her, so I wouldn’t be able to tell you exactly what all happens but all I do know is that she goes to the Aanganwadi.  Interviewer: Does anyone else go with her or does she go alone?  Respondent: Yes there are others that go with her.  Interviewer: Who else from the family goes with her?  Respondent: Either my niece or my sister-in-law goes with her.  Interviewer: Okay  Respondent: Otherwise they make a group with the neighbours and go.  Respondent: This has never happened, I send her for all the gatherings. Since my sister-in-law is there as well, I send her with her, otherwise I sometimes accompany her myself.  (Husband of 17-year-old ST primigravida attends PLA - B)  ***Socioeconomic status of family is a big contributor to ability to provide support for healthcare and education***  Interviewer: What does your husband do?  Respondent: What a poor person can do? He goes out to work.  Interviewer: What is his age?  Respondent: 18 years.  (15-year-old ST primigravida living in control area)  Interviewer: Do you go to school?  Respondent: Yes, but now I can’t.  Interviewer: When did you drop out of school?  Respondent: 8th. This month as rice harvesting has started.  Interviewer: Where does he (your husband) work?  Respondent: Bihar  Interviewer: He lives there?  Respondent: Yes he will come in March.  Interviewer: Will he go back again?  Respondent: no, he will not go back. We need to construct our home. We bought a vehicle so he had to go to earn.  Respondent: My mother-in-law also helps at times. My Husband is not here at present, he has gone to work.  Interviewer: How does your husband help you?  Respondent: he brings whatever I want to eat.  Interviewer: He is in Bihar than how does he bring?  Respondent: At present I am not having anything. How can I get anything at present? When he comes back then I will tell him what I want to have.  Interviewer: What have you thought about your future?  …Respondent: To stay good, not to fight.  ….Interviewer: What is the cause of those fights?  Respondent: Money. This or that has not been done.  Interviewer: Who fights? Who says so?  Respondent: My mother or father-in-law. Mother-in-law will say that I did not do this or that.  (15-year-old ST primigravida living in control area)  Interviewer: Your husband?  Respondent: Husband is there but he is not at home.  Interviewer: What does your husband do?  Respondent: Wall putty. (Wall paint)  (15-year-old SC primigravida in control area)  Respondent: My sister got married this year, so there is financial crisis.  (17-year-old OBC unmarried adolescent girl A attends PLA)  ***No family support due to unaccepted love marriage - No family safety net can be disastrous for a young couple***  Respondent- Like in our own family, the relations between me and my brother are not very good. When I got married they separated us from the family.  Interviewer- Oh no problem then. Since you have separated, now if she wants to study will you let her?  Respondent- [Hesitant] I don’t know if I could do it..  Interviewer- That is completely your decision. I am just asking if you would let her study if she wanted to now?  Respondent - I think of letting her study but I won’t be able to.  Interviewer- You think of letting her study but you won’t be able to? What are some of the problems? Do you think you could share?  Respondent - The problem is that I had a carrier, which was ruined after marriage. I had saved some money and had some plans that were ruined some time after I got married. I had not even been a month after my wedding that I lost everything I had. Now the condition is so that I can only eat if I go to work.  Respondent - I met with an accident after my wedding, and shortly after that my wife fell very sick. She fell so sick that for a month and a half she had a problem. She didn’t have a medical condition, but it seems she was possessed by a ghost (spirit).  Interviewer- So that brought about a lot of expenditure?  Respondent - Yes it did. We spent about 3 to 3.5 lakhs on her condition and my accident put together. And I had also gotten married recently.  Interviewer- So you paid for your own wedding?  Respondent - Yes I paid. How much could the family members give? I used to work while studying. The family members said that I was getting married to do what he could in my life, and so I thought that since I could handle my own expenditures I could get married (he fumbled around a little here so what he was trying to convey was not very clear).  Interviewer- Where do you see the two of you in the future? What are your dreams (aspirations)?  Respondent - We have dreams but we will get there slowly.  (Husband of 17-year-old ST primigravida attends PLA - B)  Respondent - We don’t live together, we live separately (from her extended family)  Interviewer- You live separately? Okay. So who lives with you here?  Respondent - Just the two of us.  Interviewer- Just the two of you?  Respondent - Yes  …  Respondent - Yes I think if my mother-in-law was around and could tell me things that would be good, but since she is not, how could she possibly tell me?  Interviewer- Your mother-in-law is not there, but is there any other woman in the village that treats you like her daughter in law?  Respondent – No, Mother-in-laws tell you what to do and what not to do..  (17-year-old ST primigravida attends PLA - B) |
| **Gossip as a mechanism for controlling and isolating girls** | In your marital home everyone is commenting on you but before marriage only our parents correct us.  (Mixed FGD C)  Respondent 1: ….The neighbors they backbite about us. They tell others that their daughter in law does not work.  Respondent 6: We feel bad about it.  Respondent 1: Say what you want on my face.  Respondent 3: Now, I know whether they are saying so for my good or bad.  Respondent 6: They backbite about us.  Interviewer: You mean they gossip more.  Respondent: Yes (Collectively)  Interviewer: Prior to marriage everything is said directly to you.  (Mixed FGD C)  Woman 1- You need to be very disciplined in the village  Interviewer- Disciplined? How so?  Woman 1- They scold you and say things about you. They say “his daughter does this and that”  Interviewer- Who do they say this to?  Woman 1- They talk among themselves.  (extended family of 18-year-old OBC mother of one)  Respondent 5: If I cook food. My husband may not say anything but my in-laws (parents) they will say it is not good.  Respondent 1: Even if they will not say then the neighbour might. “How do you cook? Learn to cook well!”  Respondent 5: “She does not cook well”. I know it is good still they will say it is not good. Like, if I did not sweep the floor then they will taunt on it too. She can simply tell me to sweep the floor but no she will say it in a bitter tone.  (mixed FGD C) |
| **Relationship with ASHA assists pregnant girls’ engagement with the health system** | |
| **PLA can enhance ASHAs role** | We got closer now. Earlier we were a bit distant. Through PLA or bond has grown strong. They themselves ask about the next meeting. They call us to ask about it. We are now a bit disturbed but they want us to conduct them early.  (ASHA FGD) |
| **ASHA invites those more easily accessible and more educated to groups** | In some villages there are educated mother and it’s easy to work with them while in other villages there are less educated and poor families women…. But still the changes are seen through PLA meetings and proper diets.  (ASHA supervisor)  There is one thing and that is. When we go to tell them about PLA. Like all the fingers are not same in the same way in the village those who are mature they understand. The ones who do not understand they say this is your work, you get paid for it. They say straight that if you will give us snacks than only we will come. This causes a lot of problem. They always say this.  (ASHA FGD)  Interviewer: Has anything good happened because of the meetings?  Respondent: The ones who try to understand, for them it is good.  (17-year-old OBC unmarried adolescent girl A attends PLA)  Respondent 1: Yes, if people are far from us (we cannot reach). Anyone can inform them about the meeting. The women next door may inform her that didi (ASHA) has informed us and we need to go in the evening.  (ASHA FGD)  Respondent 4: My house is next to Sayyia didi, she comes over and tells us. So I come even though we don’t know where she is taking us.  (FGD unmarried adolescent girls A) |
|  | **Discrepant quotes**  ***Poor relationship with ASHA***  Interviewer: She (sayiya) does nothing?  Respondent 7: She does nothing. When there is a meeting that you will find her worried. Then she takes us forcefully from whereever we are, to attend the meeting.  Respondent 4: Then she will bring her box and asks us to sign it. We do not even know the matter. She just briefs us and takes our signature and sends us. (unmarried adolescent girls FGD A)  Respondent: She should call for every meetings and help when there is any problem. Some sahiya are so that they do not come when you call them. The sahiya in my marital home, most women do not go to her. She used to call for test and then ask money for it. At home she said that it is from government and when we went there she said it costs this much give the money. This should not happen in the village. She should call for the meetings. She should give the pregnant women what is provided. She does give but then asks money for it.  (19-year-old ST secundigravida attends PLA - C)  ***Not all understand the ASHA***  What qualities should you have, that others listen to you and also participate.  Respondent: First, I should be able to win their hearts. Make them happy. Also make them realize, that what is being told is good for them. They listen to me and take me seriously. Do you know some say, “What is she saying? I did not understand it.”  (15-year-old OBC unmarried adolescent girl C) |
| **Desire to learn and develop is a strong motivator for pregnant adolescents and mothers** | On why she attends: “When I go there I get to learn things. I learn but I forget.”  (18-year-old OBC mother attends PLA)  Interviewer: Okay.... what is good about it (PLA groups)?  Respondent 2: The difficulties in becoming a mother... about it. Is there a facility to go to the hospital from the village or not. I liked that too. What are difficulties of a girl who are treated with an evil eye. That is also good information. Because this is not something to hide. We are treated in this way.  (Mixed FGD B)  How will one get this knowledge if she doesn't come to the meetings. Everyone has their own knowledge but when you come to the meetings, talk to people, discuss, then your knowledge enhances.  (18-year-old OBC primigravida attends PLA)  “Ok. These meetings that you attend. Do you think there are any changes?  Respondent: There are a lot of changes. I did not know all this.”  (19-year-old ST secundigravida attends PLA - C)  They are interested to know about themselves. Those who are pregnant they do not know anything about it. They ask questions anywhere they meet us. What they should eat or not? Whether doing a thing is ok or not? When should we go for check- up?  (ASHA 2)  Respondent: The meetings should be held.  Interviewer: Why?  Respondent: As we get a lot of information, it feels good. It is good for the village.  (17-year-old OBC unmarried adolescent girl A attends PLA)  Respondent 4: To learn new things.  Interviewer: What did you get to learn? Do you really get to learn things? I want to know that.  Respondent 1: We get to learn things.  Interviewer: What did you get to learn?  Respondent 1: Taking care of children. Protect them safe from dust. To get them treated in time.  (mixed FGD C)  ***Tools, such as games, used in PLA are helpful to enhance learning***  They said that your PLA games are very good. We will come and if we do not get time than you can conduct the PLA meeting on the day we all are here. We like PLA games. We had games in each PLA meetings so they liked it and also understood a lot of things. They said we are very happy that you are helping us.  (ASHA FGD)  Interviewer: Which of the meetings do you like?  Respondent: PLA.  Interviewer: Why?  Respondent: As everything is explained in this maybe through stories or skit. It is easy to understand.  (ASHA 3)  Interviewer: So you go because you get to learn… What else do you like there?  Respondent: I enjoy being with everyone there... Discussion about everything takes place... this and that... We also express if there is a problem.. So this discussion with everyone also feels good. This does not happen at home.  (18-year-old OBC primigravida attends PLA)  ***Most men not aware of topics in PLA groups***  Respondent (FIL) - Yes, we are aware (that they go to groups) but we do not ask what happened there.  Respondent (BIL) - We do not ask.  Interviewer: Do not ask?  Respondent (BIL) - No  Interviewer - Where have you gone? What discussion happened? Nothing like this?  Respondent (BIL) - No, we know they have gone.  ***PLA addresses the reality of our lives***  Okay.... what is good about it?  Respondent 2 : The difficulties in becoming a mother... about it. Is there a facility to go to the hospital from the village or not. I liked that too. What are difficulties of a girl who are treated with an evil eye. That is also good information. Because this is not something to hide. We are treated in this way.  (mixed FGD B)  ***We learn best together***  Interviewer: Why do you want to go? Why?  Respondent: So that I get to know things. I will hear and understand then I will think something.  Interviewer: You think that lead to something good or bad?  Respondent: I feel that such things will help me/us. It feels good to listen to things. When I went I got to hear things and got to discuss all this. Had I not gone I would not talk and get to know some new things. This is what I feel and want to go.  (18-year-old ST mother of two children no PLA)  ***Knowledge is more valuable than material goods***  Respondent: Girls do understand if we explain things to them. They are not after money.  .. Are you concerned for the child or snacks? Once we did survey too for understanding this.. it was asked by our seniors to do…  Respondent: They do reply I am concerned with my child.  (ASHA FGD)  Interviewer: Who decided that you have to go for these meetings?  Respondent: I do it myself. Sahiya didi came and told that I have to go. At home they say why do you have to go. I reply if it is my work then who else will go? So I decide and go…..  Respondent: It will benefit me if I will go. I will get to learn something from what she will tell us. When I went then she told me that we have to eat well and take care of myself.  (19-year-old ST primigravida attends PLA)  by attending PLA I knew what to eat and what not to eat.  (mixed FGD A)  ***Discrepant quotes***  Interviewer: When she does not go. Why does she not go?  Respondent: When she is busy with some work. Then she does not go. Some feel that it is useless, why should I go? We only have to hear and come back so many do not go.  Interviewer: Have you heard anyone say so?  Respondent: Yes, I have. Some say I had work here. I went there and did time pass.  (19-year-old ST primigravida attends PLA)  Interviewer: Have you done anything with the information that you got from the discussions or the meetings?  Respondent: No.  Interviewer: Do you just hear and come back?  Respondent: I have heard and came back but did not do anything.  Interviewer: What all have you heard but did not do anything?  Respondent: I did not have any problem so I did not do anything.  (19-year-old ST primigravida attends PLA)  ***Some are just not interested***  Interviewer: There might be some who have attended?  Respondent: There are just these two. They do not attend the meetings.  Interviewer: They do not come even if you ask them to?  Respondent: I even went to their home. They say they will come but do not.  (ASHA 3)  ***Adolescents are reluctant to learn***  Respondent: The main thing is that the adolescents do not want to come to Aanganwadi. They just do not come. We tell them that we want to give you some information but they just do not want to come. (Auxiliary Nurse Midwife 2) |
| **Suggestions for improving PLA groups for adolescents** | ***Enhancing participation***  Interviewer: In a group if there are aged women and also adolescent girls, who talks more?  Respondent: The aged women. The adolescents listen. The aged women say that you are doing all this now. In our time there was no such thing and we all are fine.  (ASHA 2)  Interviewer: Do the adolescent girls participate?  Respondent: They only listen. They discuss among themselves but do not talk to me directly. If they want to ask something they do so when there are no older ladies or if they meet me alone on the way.  (ASHA 3)  ***Keeping group mixed age***  Interviewer: These meetings that you attend. There are old women, women of your age, women the age of your mother and even small girls. Should the meeting of all be conducted separately or together?  Respondent: Everyone should be there.  Interviewer: Why?  Respondent: As the old will teach the young their daughter and daughter-in-law what they should do. And the young will get to know things.  Interviewer: If they will not be there and only your age girls are there? Do you not feel that if they are there you are not able to speak? Is there no such thing?  Respondent: No. It should be, we should speak.  (19-year-old ST secundigravida attends PLA - C)  Interviewer: When you go there. Many women come there. Like your mother-in-law. What do you think they all should be there?  Respondent: Yes, they should be there….  Respondent: If I did not understand but if they understood. I may not be able to understand but they do. They may explain it to me and I may understand.  Interviewer: They come and tell you?  Respondent: Yes  (18-year-old OBC mother attends PLA)  ***A space to speak freely***  Interviewer: How do you feel about us all sitting here and talking? How do you feel?  Respondent 7: It feels good.  Respondent 2: It feels good to talk about ourselves.  (Mixed FGD B)  ***Informing about the meeting***  Interviewer: If you are told that you have to conduct a meeting. than what will you do first?  Respondent 1: Inform everyone about the meeting.  (Mixed FGD C)  ***Timing***  Some are not able to attend as they have to go to school. For them we keep the meeting on Sunday at times so that they can also attend. They like it. They might be benefitting from the information given. That is why they are interested in the meetings.  (ASHA FGD)  ***Incentives***  What should we do in the meetings so that more adolescent girls come?  Respondent: They did not come earlier but since we got the projector they are coming.  (ASHA 2)  It can be improved if we serve them breakfast. (All talking together, not clear) they will think that we sat all day at least we got breakfast.  (ASHA FGD)  ***Including adolescent boys***  Respondent 4:…..The boys do not talk to us but the girls are convinced. We think that we also do not that much information to convince them. If we are taught how to convince them then if we practice it in the village, maybe they will also talk to us.  Interviewer: Do you think that you need to talk to boys of this age?  Respondent: Yes  (ASHA FGD)  ***Skilled in topics of interest to adolescents***  Respondent: At times they ask us such questions that we do not have the answers to their questions. Then we ask that to our senior and then to them in the next meeting.  Respondent 8: If we are given proper training related to all this than it will be good. We tell them that we will tell you next week but there might be things going on in her head.  (ASHA FGD)  We need to start thinking about it. Because right now we are not exactly focused on adolescent health issues.  (ASHA Supervisor)  ***Informing everyone about the meeting:***  Interviewer: If you are told that you have to conduct a meeting. than what will you do first?  Respondent 1: Inform everyone about the meeting.  (mixed FGD C)  ***Men are an untapped source of power for reproductive health of girls***  Interviewer:…. How do the men of the village help, or how do they look at her?  Husband: The men don’t tell the lady straight away, but they will tell her husband. They will ask him…why do you make her do such things?”  (Husband of 17-year-old ST primigravida attends PLA – B) |

**Exploring Adolescent Pregnant Girls engagement and empowerment through**

**Participatory women’s groups**

**GUIDELINES FOR CONDUCTING SEMI-STRUCTURED INTERVIEWS – Adolescent Pregnant Girls in PLA Groups**

***Planning and preparing for the interview***

- The interview will take around 1 to 1.5 hours
- Date and time to be decided by the mothers being interviewed
- Sitting arrangements should be such that you can see each other/preferably sit face to face

***Using the interviewing Topic Guide***

- The guide consists of a list of questions or topics to be used to keep the discussion in track from beginning to end

***Sampling and Participants***

After discussion with Ekjut it was decided that a minimum five adolescent girls who are pregnant or mothers attending PLA groups will be interviewed. They will be identified through the local ASHA Facilitator (Health activist).

Interviewees will be purposively sampled to represent at least one each from a tribal or scheduled caste group, and primigravid and non-primigravid women.

**INTRODUCE THE SESSION**

- Allow some informal discussion before the actual session starts
- Introduce yourself as the interviewer
- Let the girl/mother introduce herself
- Put the girl/mother at ease and explain the purpose of the in-depth interview, the kind of information needed and how the information will be used (Read information pamphlet)
- Ask permission to use a tape recorder and also take notes
- Don’t allow the discussion to go” off the track” - Saying: ‘wait how does this relate to…...’

Saying ‘interesting point, but how about………?’

- Conclude the session by summarising how the data will be used , ask if the girl/mother would like to add anything related to the discussion and then thank the girl/mother

**BEGINNING THE INTERVIEW**

Good Morning/Afternoon and welcome to this discussion. Thanks for giving your valuable time to share your views on the PLA groups. My name is …………………….. I am working with Ekjut on a project to better understand how adolescent pregnant girls, adolescent mothers and their babies can be better supported by the Participatory Learning in Action Groups.

You are selected for this interview because you have participated in some of the participatory learning and action group meetings. We have set questions to try to know about your perceptions and views regarding these groups and whether they helped you. My aim is to improve these groups for young mothers. Your views will be anonymous and not linked to you.

Please keep in mind that we are just as interested in negative comments as positive comments, and at times the negative comments are the most helpful.

Before beginning the session, I would like your permission to tape record the interview because we do not want to miss any of your comments. You may be assured of complete confidentiality.

Is it alright to start; if consent is given turn the recorder/Dictaphone On?

Thank you for agreeing to be interviewed. I would first like to know a bit more about you.

1. **ADOLESCENT PREGNANT GIRLS PARTICIPATING IN GROUPS**

***You and your Pregnancy/Child***

What is your age? Are you married? How long have you been married for? Do you still go to school? If not, what grade did you stop? How many months pregnant are you? Is this your first child? Who do you live with?

What are the positive and negative aspects/challenges that you have faced as an adolescent pregnant girl living here? What factors affect this? PROBE: How does your family support you during your pregnancy, in terms of work, mobility, food, education?

Where do you get your information or supplies for your sexual and reproductive health? Such as contraception? And sanitary napkins?

Some pregnant women have a low mood during pregnancy and after. Did you suffer from depression during or after your pregnancy?

***Inclusivity and Participation in Groups***

How long have you been attending women’s groups? How many times did you attend in the last 3 months?

Why did you decide to join a women’s group?

Do you have any barriers preventing you from attending the group as much as you wish? Explain.

What helps you or encourages you to attend the group? PROMPT - more young women? Support from older women?

***Impact of Intervention, including influence on empowerment, agency and social networks***

How has your experience of the women’s group been? What do you enjoy about the groups? What do you not enjoy? Do you feel close to the other members in the group?

What did you learn? How has this changed your health practices? PROMPT: antenatal visits, weight check, BP checks

What part of the group do you find more useful? PROMPT; picture card discussions, meeting others, solidarity, support within the group.

What strategies did your group identify that are helpful for you?

How do you feel about delivering a baby? Who will help you through it? What will you do if you need extra help?

Do you share anything you learnt in groups with other women and or family afterwards? Who? And what? Do you feel comfortable sharing what you have learnt?

Has the group helped you to feel that you can do things that you could not do before for your pregnancy? Like choose what food to eat? Or leave the home to ask for help?

Do you think the group has influence on adolescent pregnant girls not participating, if so how?

How does participating in women’s group affect the way the community treats you? Do you think the women’s groups helps your voice to be heard? If yes, how?

***Quality of Facilitation***

Have you ever voted to prioritise problems in a PLA group?

How many times have you spoken in a PLA group? Share examples.

What qualities and skills does a good facilitator have? How do you find facilitation of your group? How freely can you voice your own opinions? PROMPT: or do you feel shy and wait for older women or more experienced mothers to speak?

How does the ASHA help you through your pregnancy? PROMPT: visit to Auxiliary Nurse Midwife, iron tablets, home visits? Do you feel closer to her because of participating in the group?

***Outlook for the future***

How do you feel about your pregnancy? PROMPT: Are you happy to be pregnant? What are your plans for the future in terms of family?

What are your dreams for the future?

Do you have any friends you share your hopes or problems with?

Do you think there is anyone else we should speak to specifically about this topic? PROMPT: another pregnant adolescent or mother? Do you have any friends we should speak to?

How do you want to hear back about what we find in this study? PROMPT: Should we come back and share what other girls have shared?

***Is there anything else that you think would be important to mention?***

**END**

It has been really interesting to hear about your experiences and your thoughts

Thank you very much

Ask if she has any questions

Turn the recorder/Dictaphone off

# **Author Reflexivity Statement**

1. **How does this study address local research and policy priorities?**

India has high rates of early marriage and adolescent pregnancy. A community-based intervention involving women’s groups using Participatory Learning and Action to improve maternal and newborn health is being scaled up through several Indian states, including the eastern State of Jharkhand. The lead researcher (FS) did not approach local partners (Ekjut) with a pre-determined research question but only with the offer of help from a doctoral candidate interested in adolescent health. Together, and through considering many potential areas, we both determined that the focus of this study was useful, timely and feasible.

1. **How were local researchers involved in study design?**

We first gained local ethical approval through Ekjut’s Institutional Ethics Committee (IEC). The IEC has several independent members, including representatives from tribal communities, researchers, and other health NGOs in Jharkhand. IEC members were very supportive of this project and its relevance to Jharkhand, including its tribal communities. FS then spent several days with three local researchers (NN, SR, SS) to deliberate on the characteristics of respondents to interview, a meaningful and feasible sample size, as well as data collection methods and instruments. The local researchers determined and guided who would be useful to interview (e.g. Adolescent SRH worker in a hospital). They also provided insights into the reality of health care in Jharkhand. Ekjut then determined which villages we could visit, when, and with which local staff so as not to disrupt other local programs or cause undue logistical demands. All steps in the study design were closely led by Ekjut. AP has extensive experience of collaboration with Ekjut, with whom she has worked with closely for nearly 20 years. FS, although a PhD student affiliated with Melbourne University, lived in a middle-income country throughout this research project and has years of experience living or working in LMIC, including in India. Both FS and AP visited Jharkhand multiple times during the timeframe of this project, giving time for unpressured consultation and discussion. When describing the limitations of this article we acknowledge power imbalances that inevitably arise when conducting this type of research, yet not receiving any direct renumeration for this research enabled much more flexibility from FS, AP, and SM to cater to local researcher needs and priorities.

1. **How has funding been used to support the local research team?**

This was an unfunded project and part of a PhD. We conducted field visits with the Ekjut team while they were doing visits for another funded project. We thus ensured we did not hamper any of the original project’s work and workload. We sought to as much as possible minimize workload on the local research team, with FS arranging for an external translator to do the transcripts (paid personally by FS). AP, GCP, SM did not receive any funding for this research. Given research on adolescent health is a priority for Ekjut, they allocated some work time of ST and SR to assist with this research, such as the field visits and reviewing transcripts. ST also gave a lot of her personal time to assist with this research, given it would assist her in her own future planned PhD studies. ST is now pursuing a PhD, and we hope to use other data from this study to build up her publication record.

1. **How are research staff who conducted data collection acknowledged?**

FS is first author, ST second author and SR joint last author. Coordinators and Ekjut senior staff members who assisted in coordinating visits are listed in the acknowledgments.

1. **Do all members of the research partnership have access to study data?**

Yes, all members of the research partnership have access to study data.

1. **How was data used to develop analytical skills within the partnership?**

AP assisted both FS and ST to develop their capacity to code, organize, analyse and interpret qualitative data. This entailed multiple debriefing sessions, and joint reflection on themes identified. ST is now building on these skills by doing a PhD.

1. **How have research partners collaborated in interpreting study data?**

ST worked closely in interpreting the data through multiple conversations and meeting in person during visits to India (with a hiatus due to the COVID-19 pandemic). Initial identified themes were shared in a presentation and discussions with NN and SR, and their input was incorporated into the final interpretation. The paper was shared with all authors, and all authors provided their input and interpretation.

1. **How were research partners supported to develop writing skills?**

The senior authors (AP, SM) assisted the others to develop their writing skills. As this paper is part of FS’s PhD by publication the analysis and writing had to be done primarily by her. However, given the amount of data collected there are other papers in the pipeline which will be led by the research partners for which there will be more opportunity to assist in developing writing skills.

1. **How will research products be shared to address local needs?**

FS had discussions with Ekjut about disseminating findings back to ASHAs to discuss ways to incorporate adolescent-friendly training into PLA facilitator training. We also are planning to share the findings in local health consultations and with the Rural health Mission of Jharkhand,

1. **How is the leadership, contribution and ownership of this work by LMIC researchers recognised within the authorship?**

Our original ethics application had the authorship split equally between HIC (3) and LMIC researchers (3). Due to the untimely death of George Patton, Susan Sawyer stepped in as the adolescent health expert, bringing our ratio to 4:3. LMIC researchers have second and joint last authorship.

1. **How have early career researchers across the partnership been included within the authorship team?**

The first author is a PhD student, the second author was an aspiring PhD student who has since started her doctorate. The other members of the team are senior authors who guided these two authors.

1. **How has gender balance been addressed within the authorship?**

Except for one author, all the authors are female. As stated in the paper, culturally and as advised by the local ethics committee the interviewers were required to be female.

1. **How has the project contributed to training of LMIC researchers?**

ST gained experience in developing a research article from beginning to end. This has been very helpful for her future research career plans. Throughout this research we also assisted her with her other research projects. ST will be drawing upon this project to hopefully win sponsorship in a research program for a year targeted to LMIC emerging researchers. The two other LMIC researchers on this paper are experienced and senior researchers.

1. **How has the project contributed to improvements in local infrastructure?**

The project did not contribute to improvements in local infrastructure.

1. **What safeguarding procedures were used to protect local study participants and researchers?**

The project was first assessed by a local ethics committee. Further input was provided by Melbourne University ethics. During very initial discussions with Ekjut senior staff, we determined and clarified a practical distress protocol, which had clear exclusion criteria. There was checking first with the ASHA about the suitability of persons to interview, and no person was interviewed without full and willing consent. Timing of all visits to Jharkhand by FS and AP were done to fit with partners’ availability. Nonetheless, we acknowledge that it took considerable effort to accommodate this study in addition to their already busy and full schedules and appreciate the support and encouragement they provided to this work.
